# Farmers’ and stakeholders’ views on the adoption of agroecological practices. Results from participatory workshops in European countries

**DOI:** 10.12688/openreseurope.20536.2

**Published:** 2025-12-11

**Authors:** Jacques-Aristide Perrin, Alexander Wezel, James Henty Williams, Bettina Wenzel, Hella Kehlenbeck, Aurélie Ferrer, Gordana Đurić, Gvozden Mićić, Dimitrije Markovic

**Affiliations:** 1 Agroecology and Environment Research Unit, ISARA, Lyon, France; 2 Rural Studies Laboratory, ISARA, Lyon, France; 3 Department of Agroecology - Agricultural biodiversity, Aarhus University, Aarhus, Denmark; 4 Julius Kühn-Institut Federal Research Centre for Cultivated Plants Institute for Strategies and Technology Assessment, Kleinmachnow, Brandenburg, Germany; 5 Poljoprivredni fakultet, University of Banja Luka, Banja Luka, Bosnia and Herzegovina; 6 Agrobiodiversity & Agroecology, Alica Foundation, Banja Luka, Bosnia and Herzegovina

**Keywords:** Agroecology, farmers’ motivation, environmentally friendly practices, farmers’ knowledge, stacking of practices

## Abstract

Agroecological practices are prerequisites for many agri-environmental schemes in Europe and are increasingly being integrated into the EU’s Common Agricultural Policy. Against the backdrop of recent protests by farmers in many European countries, with various grievances based on perceived tightening of environmental regulations and economic pressures, these protests raise questions about how farmer perceptions might hinder the adoption of agroecological practices. A series of workshops with farmers, farm advisors, and other supply chain stakeholders were organized between 2022 and 2023 in different European countries to facilitate knowledge sharing and to learn farmers’ and stakeholders’ views. Exercises were conducted during the workshops to explore the extent to which farmers were adopting practices that directly or indirectly promote biodiversity. The third and final phase aimed to better understand the motivations, needs, and potential trade-offs associated with implementing different agroecological practices, as well as their combination to promote functional biodiversity. The active participation of stakeholders was beneficial for collectively reflecting on relevant agroecological transition pathways, particularly farmers rational for implementation of agroecological practices for more sustainable agriculture in Europe. The results highlighted that participants had relatively good knowledge of agroecological practices and an inherent understanding of their benefits. Future new practices considered by the participants were mainly intercropping, the use of organic mulches, and direct seeding. Currently used practices, most often combined with others, include diversified crop rotations, biocontrol, and cover crops. The two main combinations were ‘diversified crop rotation - cover crops’, and ‘biopesticide-biological control’. The main reasons for implementing different practices were that these practices are easy to implement and inexpensive, have a proven track record of success, or were subsidized. The main existing and potential future barriers to the implementation of agroecological practices were identified as lack of knowledge, appropriate financing, market opportunities, and lack of information. To overcome these obstacles, the strategies discussed in this article can facilitate or expand the implementation of agroecological practices. This work may thus be of interest to decision-makers in determining how to consider the views of farmers and technical advisors in developing policies.

## Introduction

Farmers in Europe need to fulfil multiple functions ( Schröder *et al.*, 2020). On the one hand, they produce food and other agricultural products for European populations, exports, and provide jobs. On the other hand, they are expected to contribute to the conservation of rural landscapes and biodiversity ( Estrada-Carmona *et al.*, 2022), and limit agriculture's contribution to climate change. As a set of farming practices, agroecological practices ( Wezel *et al.*, 2014) and their enhanced use can play an important role in supporting these expectations in mitigating the negative environmental impacts associated with conventional or intensive agriculture and support biodiversity such as establishing hedgerows or planting flower strips. Through the optimization of natural processes, these practices provide various ecosystem services known for soil organic matter improvement, nitrogen fixation, soil conservation, weed and pest control, pollination services, biodiversity conservation, and water purification ( Albrecht *et al.*, 2020; Skaalsveen & Clarke, 2021).

Despite their central position within agri-environmental schemes in Europe, where they are increasingly integrated into the EU’s Common Agricultural Policy ( Langlais, 2023; Linares Quero *et al.*, 2022), some agroecological practices are still only marginally adopted by farmers ( Rizzo *et al.*, 2024; Thompson *et al.*, 2024). This low uptake of agroecological practices raises questions about farmers’ perceptions and motivations in implementing different agroecological practices to help explain the reluctance to change practices as well as potential barriers and constraints ( Dumont *et al.,* 2021; Maas *et al.*, 2021). In this respect, the adoption of sustainable farming practices has already been analyzed in different contexts ( Han & Grudens-Schuck, 2022; Mićić *et al.*, 2022; Serebrennikov *et al.*, 2020) such as participation in agri-environmental measures ( Canessa *et al.*, 2024). However, there is still limited information on the adoption of agroecological practices.

Moreover, widespread uptake of agroecological practices is set against the backdrop of recent protests by farmers in many European countries due to socio-economic pressures and a sense of onerous environmental regulations ( Finger *et al.*, 2024), and a reluctance to voluntarily embrace ‘environmentally friendly measures’ ( Anougmar *et al.*, 2025). Such underlying socio-economic issues and perceptions among farming communities, about lack of recognition of farmers’ practical experiences ( Coolsaet, 2016; Markiewicz-Keszycka *et al.*, 2025) raise questions about the contribution of agroecological research and proposals for new ‘environmentally friendly’ production models and their uptake among farmers. A possible mismatch between their expectations and new agroecology-oriented research needs to be questioned to identify the most important criteria leading farmers to use the knowledge generated. It could be helpful to understand what kind of agroecological knowledge is preferable to satisfy agricultural stakeholders, such as producers, supply chain actors, and technical advisors.

To investigate and understand the present knowledge gaps and possible discrepancies regarding the adoption of agroecological practices, we present an example of a participatory research approach that involves representatives of different stakeholder groups, who, in various ways, influence farming practices e.g., from farmers to policy makers. Stakeholder engagement, with a wide and diverse range of actors, is a useful way of collectively exploring perspectives and gaining practitioner insights, which were pertinent to the promotion and adoption of agroecological practices. The engagement of a broader set of stakeholder groups was intended to gain a better understanding of the broader context of their decision making.

Our study has two main objectives. The first was to gain insights into the views and usage (exploring benefits/barriers) of agroecological practices among farmers and stakeholders, as well as their reasoning for using a particular practice. The second objective was to discuss the possibilities of combining individual agroecological practices, termed ‘stacking’. The concept of ‘stacking’ agronomic practices was recently developed and described by Lehman *et al.* (2019). It is defined as the addition of one or more “sustainable” practices to a simplified agroecosystem to maximize positive effects. The application of multiple practices in an agroecosystem can create synergies ( Christianson *et al.*, 2018) which improve the effectiveness of ecosystem service provision and the diversity of services supported. The resulting effect of combining two or more practices can be greater than the simple addition of the effects of the practices taken separately ( Lehman *et al.*, 2019). However, little is known about the real efficacy of stacking multiple sustainable practices in agroecosystems, or practical aspects faced by farmers currently implementing them, along with the constraints and barriers to implementing these practices. To prescribe effective stacking strategies, it is necessary to better understand the underlying reasons and interests of farmers in using individual and combinations of agroecological practices, as well as the expected benefits and barriers.

## Materials and methods

### Workshops and profiles of participants

Eleven workshops with farmers, farming advisors, and other associated stakeholders (see Table 1) were held in nine European countries between 2022 and 2023. The workshops were organized by nine national research teams involved in the Horizon 2020 EcoStack project ( https://ecostack-h2020.eu/). The aim of this research project is to develop ecologically, economically and socially sustainable crop production strategies via stacking of functional biodiversity. A specific objective was also to study farmer perspectives regarding a shift in focus from implementing single farm management practices to ‘stacking’ different practices. While benefits of agroecological practices are widely known, little information is available on combining those practices.

**Table 1.** Number of workshop participants, participant profiles and location of the workshops.

Country	Number of workshop participants	Profile of participants (number by profiles)	Date
**Bosnia and Herzegovina**	34	Farmers (24), farm advisors (2), local authorities (2), students (6)	March 2023
**Bulgaria**	18	Farmers (13), NGO (2), teachers (3)	March 2023
**England**	12	Farmers (12)	March 2023
**Finland**	15	Farmers (15)	February 2023
**France**	7	Farm advisors (7)	February 2023
**France**	13	Farmers (6), farm advisors (7)	March 2023
**Germany** **(Lower Saxony)**	7	Farmers (7)	February 2023
**Germany** **(Hesse, online)**	136	Farmers (67), students (29), farm advisors (25), scientists (13), others (2)	February 2023
**Italy**	17	Farmers (10), advisors (3), association (2), teachers (2)	March 2023
**Portugal**	14	Farmers (5), advisors (6), regional government officials (3)	September 2022
**Spain**	13	Farmers (6), advisors (2), regional government officials (5)	September 2022
**Total**	286	Farmers (165), advisors (52), students (35), scientists (13), regional government officials (8), teachers (5), NGO (2), association (2), local authorities (2), others (2)	

The countries in which the workshops took place are the countries of the research teams involved in this project. All partners used a common protocol, specifying different workshop phases with several exercises as well as questions asked to participants. All participants were contacted in advance by e-mail, are volunteers, and signed a written consent form outlining the project's objectives, guaranteed anonymity and the recording of workshop exchanges.

The first phase of these workshops was to present selected EcoStack research findings about selected agroecological practices, such as cover crops and cultivar mixtures, relevant and tailored for participants attending in different countries. The second phase was used to explore the extent to which farmers adopted practices that directly or indirectly supported biodiversity. The third and final phases sought to gain insights into the motivations, needs, and potential trade-offs in implementing different agroecological practices, as well as their stacking to promote functional biodiversity. The duration of the workshops was between three and five hours, depending on the number of participants.

A total of 286 participants attended the eleven workshops, the majority were farmers (165). With the exception of Bosnia-Herzegovina and Germany, a similar number of participants took part in each workshop. All participants were selected on the basis of of their professional profiles. The participating farmers and stakeholders were selected by project research teams based on their experience with different farming systems (cereal crops, root crops, horticulture, mixed crop-livestock) and the cultivation of relevant crops for the EcoStack project. Both organic and conventional farmers participated in the workshops, although the proportion of organic to conventional farmers varied from workshop to workshop. For example, in Bosnia-Herzegovina, only three of 23 participants described their farm as organic (non-certified production). Conversely, in Germany for both workshops, around three quarters of participating farmers described their farm as organic.

### Description of the phases of workshops regarding agroecological practices investigated

Through this study on practices, we consider agroecology as a dynamic and holistic approach including made up of science, implementation and management of agroecological practices and transition to sustainable and fair food systems. Our work is based on a mixed-methods approach that allows us to study the interactions between agricultural practices, public policies, and social dynamics involved in the agroecological transition. This paradigm is particularly well-suited to analyzing the perceptions and experiences of farmers and advisors, and to understand how these elements influence their decisions and trajectories. The agroecological practices ( Wezel *et al.*, 2014) targeted within the workshop were intercropping, cover crops, no-till, biocontrol agents or biopesticides, cultivar mixtures, organic mulches, diversified crop rotation, local crop varieties, direct seeding, and off-crop elements that support biodiversity, such as hedgerows or flower strips (see Table 2).

**Table 2.** Agroecological practices investigated and their characterisation as defined by Wezel *et al.*, 2014.

Name of the agroecological practice	Short characterization
*Intercropping*	Mixture of two or more crops, also undersowing
*Cultivar mixtures*	Mixture of two or more cultivars
*Cover crop*	Secondary crop cultivated between two main crops, harvested or not (green manure)
*Organic mulches*	Straw mulch or other organic material is spread between the crop rows
*Diversified crop rotation*	Succession of four or more crops on the same field
*Off-crop measures/Landscape elements*	Semi-natural landscape element such as hedges, tree lines, thickets, ponds etc.
*Local crop varieties*	Use of locally adapted and available crop varieties
*Direct seeding*	Seeding without pre-tillage (directly into a cover crop or mulch)
*Biological control*	Use of natural enemies for reducing or mitigating pests
*Biopesticides*	Pesticides based only on natural ingredients, e.g. Bacillus thuringiensis
*Grass strips*	Grass strips inside a field or on borders of a cropped field
*Flower strips*	Flower strips inside a field or on field borders

Several exercises were carried out with the participants in which they were asked to respond to specific questions. Data sources included responses to these structured exercises on the adoption of agroecological practices, written workshop materials (flipcharts, ranking tables), and facilitator notes. The first exercise was to determine an inventory of the agroecological practices amongst participants. To do this, each participant was invited to choose from the list of practices ( Table 2), to indicate what they currently use and what they might consider implementing in the future. Online participants in Germany were also able to answer these questions by voting remotely. Each participant could choose as many practices as they wanted. The choice concerning the future had no commitment value: it was a thoughtful intention.

The second exercise was to identify the perceived challenges in adopting listed practices. To identify obstacles to adopting agroecological practices, we asked participants to write post-it notes answering: (1) why do farmers not use certain practices? (2) What encourages farmers to use them? Not all practices were necessarily covered in the second exercise of the workshops, as they depended on the profile of the participants and whether they were already using them.

In the third round, the participants were asked to indicate the main reasons that would make them more inclined to implement agroecological practices. Participants were asked to choose their top-3 from ten suggestions ( Table 3) that had been previously identified by the research team. Of the three votes, only one per participant could be attributed to one reason for adoption. However, they also had the option of adding it to the list on post-it notes.

**Table 3.** Pre-selected reasons given by the authors to participants for adopting agroecological practices.

Reasons for adoption of practices
1. Subsidies
2. Proven results
3. Easy to implement
4. Low cost
5. Improved ‘natural’ plant protection
6. Biodiversity conservation
7. Fit with existing production system
8. Good image for farmers
9. Support from advisors
10. Others

**Figure 1.**
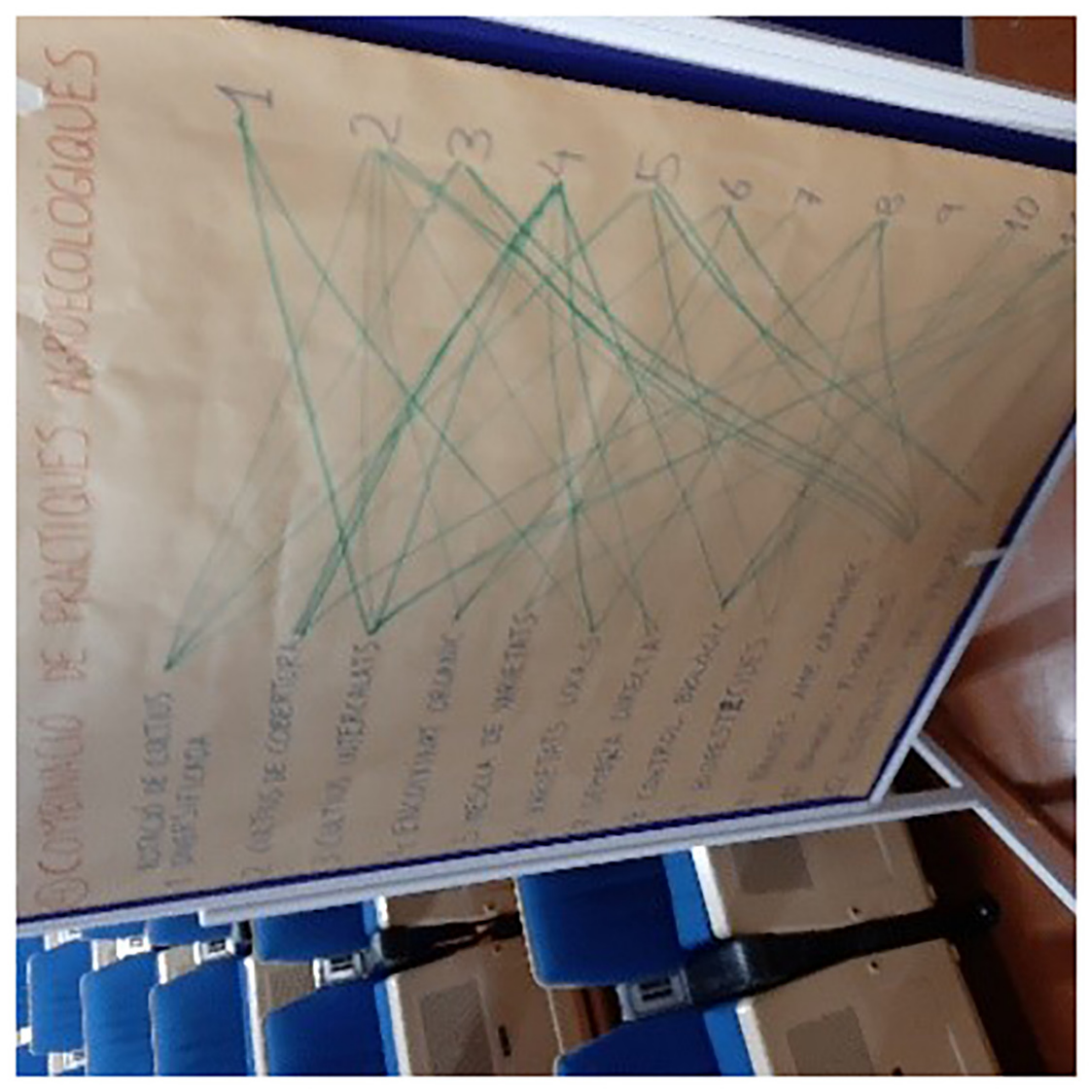
Illustration of the large panel for exercise no.4 in Spain/Catalonia.

The final exercise consisted of making an inventory of the combination (stacking) of practices and understanding the potential benefits as well as constraints and barriers to combine agroecological practices. For this, each participant was asked to link on a board the practices they were combining ( Figure 1). The twelve practices were listed on the left of the board, with numbering from 1 to 12 and full statement of the practice, and listed in the same order (using only their number) on the right of the board. In this way, the participants were able to graphically and visually represent the combinations of two practices. The individual printed boards were then collected and displayed together on a larger board to give an overview of the different responses and to launch the discussion. This exercise was only carried out in five countries (i.e. Bosnia and Herzegovina, Finland, France, Italy, Spain) due to a lack of time and sometimes lack of relevance, depending on the profile of the participants. It is important to clarify that the participants interpreted this question as follows: what practices have you already combined? Although the farmers’ response was meant to demonstrate that they had already done this, it did not imply that they did it on a regular basis. The cooperative advisors' responses were informed by their understanding of the cooperative members' practices.

Two main types of data were generated. First, quantitative data consisted of scoring of current and anticipated use of agroecological practices, rankings of practice combinations, and frequency counts of perceived benefits and barriers. Second, qualitative data were derived from transcribed discussions, including participants’ statements and arguments.

The following results were derived from the outcomes and reports of the different workshops, which focused on the barriers that hinder the transition to more agroecological farming, in our case in relation to various practices that contribute to crop protection. Not all 286 participants took part in all these exercises, which were designed to give a voice to farmers responding to their own operations and practical experiences, as well as to advisors supervising and/or advising farmers. Only 146 took an active part in the exercises because the others stated that they did not feel sufficiently concerned or legitimate to respond. As the questions were mainly addressed to farmers and advisors providing services to farmers, other participants were unable to answer questions on practices. However, they took part in discussions arising from these exercises.

A mixed qualitative–quantitative analytical approach was applied. Descriptive statistics were used to identify the most frequently implemented agroecological practices and practice combinations, thereby helping to characterize general trends across workshops. In parallel, a thematic content analysis was conducted to identify motivations for adopting specific practices, perceived barriers, and knowledge needs.

## Results

### Participants’ present and prospective use of agroecological practices

In workshops held in several European countries, participants were asked to indicate which practices they already used and which additional practices they would like to introduce in the future. Table 4 summarizes the current and intended future use of agroecological practices.

**Table 4.** Current use of different agroecological practices and intended future use according participants (n=146). Multiple responses were possible to indicate current or future use of practices. Summary of answers from different European countries (Bosnia and Herzegovina, Bulgaria, England, Finland, France, Germany, Italy, Portugal, Spain).

Agroecological practice	Current use of practice		Considered future use of practices	
	No. of responses	% of the total number of votes (rounded)	No. of responses	% of the total number of votes (rounded)
**Diversified crop rotation**	39	15	7	5
**Integration of landscape elements**	35	13	12	8
**Cover crop**	33	13	14	9
**Direct seeding**	25	10	16	10
**Intercropping**	20	7	18	12
**Biopesticides**	19	7	13	8
**Flower strips**	19	7	13	8
**Biological control**	18	7	12	8
**Grass strips**	16	6	10	7
**Organic mulches**	13	5	18	12
**Local crop varieties**	13	5	13	8
**Cultivar mixtures**	10	4	8	5

The three practices most commonly used practices (more than 10%) are currently diversified crop rotations, the integration of semi-natural landscape elements (e.g. hedgerows) and cover crops. These are followed by direct seeding and intercropping. Among the new practices that farmers intend to use on their farms in the future, intercropping, the use of organic mulches and direct seeding were mentioned by more than 10% of farmers. The techniques that farmers most commonly cited as now in use aside from cover crops were only partially listed as potential future uses.

### Participants’ perceptions of the benefits and barriers to implementing agroecological practices

The participants identified different advantages and constraints regarding the use of some of the selected agroecological practices. The results are presented in Table 5, summarizing the common themes from all countries. We classify each benefit and constraint using a color scheme. While the green color corresponds to environmental services, the black color refers to the technical management of the farm, and the orange color indicates economic dimensions. The advantages and constraints of each practice are dependent on a variety of contextual factors: the specific soil and climate conditions, the state of soil fertility in the fields, the choice of a technical itinerary, the availability of the equipment needed for the practice, the cost and time required for practices, the market’s demand for selling the crops, and the size of the farm. In addition, the role of knowledge production and transfer is also important in explaining the ideal conditions for the successful implementation of a practice (e.g., selection of cultivars to be combined, soil preparation, ideal chop length of the mulch material, and the spreading width between the crop rows).

**Table 5.** Advantages and constraints for implementation of agroecological practices as stated by workshop participants in different European countries (Bosnia and Herzegovina, Bulgaria, England, Finland, France, Germany, Italy, Portugal, Spain).

Practice	Advantages	Constraints
**Diversified crop rotation**	- maintenance of soil fertility - reduced presence of weeds and diseases - easy to implement	- not possible to specialize in a single crop
**Cover crop**	- prevent nutrient leaching - keep soil moisture - prevent weed development - produce fresh biomass as green manure	- under drought conditions, the cover crops do not germinate and establish well - requires a special seeding machine for sowing cover crop seeds which may differ from those of the usually cultivated plants - seeds are expensive
**Intercropping**	- choice of different crops - increased diversity in cropped area - input of nitrogen (N-fixation) if legumes intercropped - can enhance weed control - improved soil cover - attracts natural enemies of pests - undersown crop can be used as fodder for livestock - improved workability for heavy soils - limitation of fertilizer need	- some climates and weather patterns may not be suitable - complicated because the crops have different requirements for the soil - different maturation times of mixed crops rending the harvest more challenging than with pure crop - weed management more difficult - fertilization might become trickier as different crops have different needs - the harvest can be difficult and more field traffic may be required - lack of knowledge about the best combinations (various cereals with leguminous plants) - processing costs of the harvested mixed crops (sorting, sieving) - may not be economically successful because the global market doesn’t value the products - farmers may not have enough land (two cash crops mean dividing the yield of each crop by 2. Small yields are more difficult to make profitable in terms of marketable volume and storage)
**Cultivar mixture**	- useful for improving protein concentration in cereals - getting higher yield - mitigates stress and disease and reduces pest populations - limits problems due to freezing for early maturing varieties + lagging pattern of the ‘normal’ cycle of cropping because of climate change - can be interesting and used only for the production of animal feed (pigs or chickens) at small scale farms - limitation of financial risks/stabilize yield - limitation of fertilizer need	- different maturation times create problems as cultivars grow differently - difficulty and complexity to mix the cultivar (role of mechanization) - needs infrastructure to separate the seeds - needs highly skilled people for guidance - may be difficult in sale because farming contract requiring a pure variety to ensure a steady supply with the same product (authorisation/ban of cultivar mixture because of the rules of the global model/milling industry such as forbidden in the catalogue) - seed costs and difficulty to have the adequate seeds (certified seeds in organic agriculture) - lack of tools and additional costs related to sowing (outdated machinery that needs to be renewed).
**Organic mulching**	- positive influence on water availability in soils and maintenance of moisture in the soil clear effects on soil erosion reduction - plants are growing better - weed control - reduction of aphids, virus transmission - low cost of implementation of organic mulch for the organic farmers because they collect the harvest residues or fresh grass at their own farms	- fear on soil compaction - distribute mulch in the bigger fields without specialized machinery, requiring new investment - increased fuel consumption due to higher use of additional machinery or payment for intervention of provider/contractor - high costs related to production or mulching material for the conventional producers - time-consuming
**Off-crop measures** **(including flower strips, grass strips, hedgerows)**	- attract pollinators for crop pollination and natural enemies for pest control (shelter for beneficial insects and wildlife) - improve aesthetic value of the farm and landscape - natural barrier on the farm (landscape separation for human movement)	- vegetation structures (such as perennials trees or shrubs) are shading the crops (+ trees use water) - expensive establishment (flower strips, grass strips, hedgerow) - time-consuming for maintenance - takes away productive area - lack of appropriate flower and grass mixes suitable for local conditions

### Participants’ main reasons for adopting agroecological practices

The top three reasons for adopting agroecological practices (three possible votes per participant) are presented in Table 6. The most important reasons for adoption were the granting of subsidies for these practices, proven effectiveness, ease of implementation, and low costs. In a further discussion, workshop participants were asked about the main existing and possible future barriers to the implementation of agroecological practices. They identified a lack of knowledge, appropriate financing, market opportunities, and information.

**Table 6.** Ranking of the most important reasons for adopting agroecological practices as stated by participants (n=146) in workshops in EU countries (Bosnia and Herzegovina, Bulgaria, England, Finland, France, Germany, Italy, Portugal, Spain).

Reason	Absolute number of votes	% of the total number of votes (rounded)
Subsidies	24	15
Proven results	22	13
Easy to implement	22	13
Low cost	20	12
Improved ‘natural’ plant protection	18	11
Biodiversity conservation	15	9
Fit with existing production system	15	9
Good image for farmers	14	8
Support from advisors	9	5
Others	6	4

### Main combinations of practices used by participants

The stacking strategies of agroecological practices must be tailored to the respective bio-physical and agroecological contexts. To understand the overall benefits of stacking, it is necessary to focus on synergies where achievements in one goal contribute to advances in other ecosystem service delivery goals and agronomic techniques.

Two combinations were common to all five countries (Bosnia and Herzegovina, Finland, France, Italy, Spain) where this stacking exercise was carried out: (1) ‘diversified crop rotation and cover crops’ and ‘biopesticide-biological control’ and (2) ’diversified crop rotation and direct seeding’ and ‘cover crop and direct seeding’. The practices most frequently stacked with the others were diversified crop rotations, biocontrol, and cover crops. Although trends emerged in the stacking of practices, the percentages of all combinations emphasized their importance. In fact, the first combination identified by participants, 'diversified crop rotation and cover crop’, was mentioned 18 times but represented less than 10% of all combinations identified. Diversified crop rotation, biological control, and direct seeding appear to be the most suitable practices for farmers to combine at the farm level. In Table 7, the combinations that were never mentioned by the participants are shown in light gray.

**Table 7.** Results of the workshop exercises on stacking. Coloured practices are the most common practices used in combination in all countries together. The numbers indicate mention by a participant. Summary of answers from different European countries where stacking exercise carried out, Bosnia and Herzegovina, Finland, France, Italy, Spain.

	DCR	CC	OM	IC	CM	LCV	DS	BC	BP	GS	FS	LE
1. Diversified crop rotation (DCR)												
2. Cover crop (CC)	**18**											
3. Organic mulches (OM)	4	6										
4. Intercropping (IC)	3	7	1									
5. Cultivar mixtures (CM)	6	0	4	1								
6. Local crop varieties (LCV)	4	2	1	0	**7**							
7. Direct seeding (DS)	**13**	**13**	1	**7**	2	1						
8. Biological control (BC)	5	0	1	2	0	1	2					
9. Biopesticides (BP)	0	0	0	0	1	1	1	**16**				
10. Grass strips (GS)	2	3	0	3	0	1	0	**7**	0			
11. Flower strips (FS)	0	1	2	0	1	0	0	6	1	2		
12. Landscape elements (LE)	1	1	2	0	3	2	0	6	0	2	**7**	
**Total number of mentions per practice**	56	33	12	13	14	6	3	35	1	4	**7**	

## Discussion

### Farmers use of practices

In relation to these contextualized results derived from the participants’ profiles and the exercises conducted, the first observation was that the participants’ awareness and knowledge of agroecological practices was growing. They are increasingly familiar with agroecological approaches and the inherent benefits of enhancing natural processes and ecosystem service provisioning (e.g., soil fertility, pest control, and pollination), even if they are not fully aware of the concept of agroecology. There was a striking contrast in the perception of agroecology between the participants and the research teams. Indeed, the terms used by both to which the practices refer do not come from the same frame of reference between science on the one hand and the administrative context that financially supports the practice on the other. Scientific research teams take a fairly systemic and comprehensive view of agroecological practices, focusing on ecological interactions, while farmers adopt a more practical perspective related to the terminology of CAP echo-schemes. Most eco-regime measures are based on greening obligations that contribute to the eco-efficiency and sustainability of their operations ( Runge *et al.*, 2022; Zieliński *et al.*, 2025).

Introducing the topic of agroecology through the approach of practice and drawing on their practical experiences is a relevant way to discuss with participants, particularly farmers. The most common practices among respondents (i.e., crop rotations, integration of semi-natural landscape elements, and cover crops) are consistent with those reported in the European literature ( Palomo-Campesino *et al.*, 2018; Rega *et al.*, 2022; van der Ploeg *et al.*, 2019). The participants recognize some need to change their practices, some to a larger extent, and others to a smaller extent. Respondents' perception that they are already using these practices and have no plans to alter their cropping systems may be one of the reasons why they are less commonly mentioned when considering the future. As our results show, many who have started with agroecological practices have already taken the plunge of financial support and proven knowledge. However, these results depend on the country (socioeconomic conditions and available funding) and the type of crop produced by the participants. This is in line with Linares Quero *et al.* (2022), who worked on levers that hindered agro-ecological farming systems. Common Agricultural Policy payments, national regulations (country-specific policy), and transfer knowledge where the farms are located significantly affect the number of practices, indicating that policies promoting agroecological practices can have an effect.

### Constraints and barriers for implementation of practices

As with another design workshop initiative ( Jeuffroy *et al.*, 2022), the exercises and discussions in the workshops allowed us to understand the participants’ choices for adopting agroecological practices and to better understand the appropriate strategies for increasing adoption or encouraging the testing of these practices. By analyzing participants' responses, both in terms of the advantages and disadvantages of their practices, and in terms of levers, scaling up agroecological practices entails surmounting a number of sociopsychological ( Bakker *et al.*, 2021; Wiedemann et Inauen, 2023), technical and institutional barriers ( Barnes *et al*., 2022). It would be difficult to promote agroecology without addressing cognitive ( Weituschat *et al.*, 2022), technological ( Plumecocq *et al.*, 2018) and supply chain ( Kuokkanen *et al.*, 2017) lock-in effects. For example, most conventional farmers among the participants pointed out that the introduction of new agroecological practices should contribute to increasing crop yield and farm income, whereas for organic producers, it is important that they do not have negative effects on the environment. There is great tension between productivist thought and the post-productivist agricultural regime ( Wilson, 2001) which continues today. An agroecological transition has to give meaning to the future of agriculture and a clear societal purpose to support the formation of a new farmer identity ( Letourneau & Davidson, 2022) with renewed risk perception ( Eitzinger *et al.*, 2018) and renewed habitus ( Stotten, 2021). Some farmers may be resistant to change or feel pressure to conform to traditional farming practices. The cost of adopting agroecological practices is also important, particularly in terms of the potential impact on farm income. In this respect, knowledge of the costs of improving functional biodiversity at the farm level is still limited despite recent work ( Wenzel *et al.*, 2024). The introduction of a financial tool and external payments ( Zabala *et al.*, 2017) to support transition and stacking would enable the identification of gradual and adaptive transformation paths for farmers.

Farmers’ attitude toward risk ( Iyer *et al.*, 2020) is a factor to be considered because farmers may worry about the lack of skills needed to manage the fields or the effect of uncertainty, but also about the absence or low level of risk sharing between upstream and downstream sectors. As the literature on the subject shows, production contracts ( Jouan *et al.*, 2019) play a key role in taking constraints and uncertainties into account. In addition, in relation to risk mitigation through compensation, there is evidence that farmers are reluctant to voluntarily adopt voluntary agri-environmental measures and schemes, especially where environmental outcome conditions ( Anougmar *et al.,* 2025). The discussions during the workshops showed a gap between the expectations of scientists and the risk aversion of farmers with regard to the extension of agroecology, which requires different strategies.

### Interrelated strategies for enhanced implementation of agroecological practices overcoming barriers

Gaining insights from the workshops, we identified potential strategies for farmers and advisors. We consider a ‘strategy’ as a plan of action designed to achieve a medium- or long-term goal, such as the implementation or widespread use of the identified agroecological practices. These strategies serve as pillars for increasing or easing the adoption of agroecological practices.

There is a need for holistic integration of agroecological practices at the farm level, but there remains a gap between these individual practices and the combination of practices between them, which calls for holistic thinking at the farm level. Thinking about stacking, be it in time or space, implies changing scale (from plot to farm), linking both production and the environment, and moving away from reductionist logic. Participants combine these practices, either on their farm or in the same field, but they still tend to think of it as isolated practices or as something to add up. However, they do not see it with a holistic approach or synergistic benefits for the provision of ecosystem services at cropping systems and farm scales. Most of them could not envision agroecology being implemented on their farms holistically. This shows the persistence of silo thinking that segments and isolates individual practices rather than recognizing their interdependence.

Improved understanding of practical and beneficial aspects of stacking agroecological practices: Our research reveals an initial difficulty in thinking in terms of stacking. As farmers were unaware of the impacts and potential synergies of some agroecological practices, they chose or preferred measures that they thought they could combine, whose impact they thought they knew, and for which they had machinery to implement. It is not possible to exchange views on the benefits of stacking, such as additive, synergistic, antagonistic, or neutral, as suggested by Lehman *et al.* (2019). Therefore, questions remain regarding what motivates farmers to combine different agroecological practices. For example, do participants combine practices with the purpose of promoting synergies between the practices, biodiversity conservation, and ecosystem services provision, or is the combination simply an effect of the management strategy that suits the farm socio-economic and bio-physical environment? Is this justified only on a field basis? Can there be antagonistic or neutral effects of this combination from the farmer’s perspective? Stacking strategies must consider trade-offs between combinations of practices in more complex agroecosystems. It was not possible to reach this level of discussion because of the profile of the farmers in terms of their progress towards agroecology, their lack of awareness of the subject, and the time available during the workshop.

Fostering multi-scale learning through transdisciplinary approaches to agroecology: Farmers, advisors, and scientists need to learn about the complexity of the agroecosystem and its relationship with the ecosystem, so that they can gradually learn to think in a multi-scale and systemic way. To achieve concrete results, this learning cannot merely be theoretical or conceptual. It requires the implementation of transdisciplinary co-designed projects capable of evolving the skills and practical experience of different stakeholders, such as farmers, advisors, and scientists. Therefore, a more in-depth study is needed to understand the full complexity of any synergy between agroecological practices. To illustrate this state of thinking in relation to stacking, we propose avenues of research that should be explored in more depth in this subject ( Table 8). These research questions were based on the discussion outcomes with farmers in the workshops.

**Table 8.** Challenges of research to be explored to gain a more comprehensive understanding of stacking strategies related to the four practices combinations most frequently mentioned by farmers in workshops.

	Challenges			
Most mentioned practice combinations by farmers	Agronomic dimension	Socio-economic dimension ^1^	Effects and observed by scale of analysis (plot, farm)	Assessment of type of benefits of stacking
**Diversified crop rotation – cover crop**	How to assess both the impact of crop management measures and soil and water-related measures, and their effects in terms of yield?	How to assess what stacking generates in terms of capital investment, labor demand and requirement, profitability, as well as resilience and autonomy for a farm?	How the local factors of a farm interact with several spatial scales, perhaps even modifying the overall structure of the landscape, with the aim of measuring ecosystem services?	How to evaluate the synergies and need for trade-offs of different agronomic management depending on the combination of practices, to be able to make recommendations?
**Diversified crop rotation - Direct seeding**				
**Direct seeding - cover crop**				
**Biopesticides - Biological control**				

^1^ Inspired from Mouratiadou *et al*., 2024

The avenues of future research in Table 8 propose carrying out a multi-criteria assessment (technical, environmental, socio-economic) to understand what the practice of stacking can generate in terms of both knowledge and interest for the sustainability of farms. Knowing these data in order to launch an analysis will make it possible to determine how the risks associated with stacking practice can be shared and how researchers can help to supervise and support this form of transition.

This type of transdisciplinary project based on the co-design of practices ( Barrios *et al.*, 2020; Berthet & Hickey, 2018) could involve farmers, researchers, and other stakeholders. It is a path considered desirable by the literature for ethical reasons ( Berthet & Deroche-Leydier, 2022), to facilitate the transfer of results to a given field, to consider situational knowledge and learning processes ( Ensor & de Bruin, 2022), for its creative potential ( Prost *et al.*, 2017) and its inclination to change. A framework of mutual trust ( Stallman & James, 2017) to promote dialogue and companionship would provide a strong incentive to develop agroecology. It is worth noting that participants called for better ‘networking’ across different levels (e.g. individuals and colleagues), meso-level (e.g. communities and organizations) and macro-level (e.g. legal systems and food supply chain) that together shape management decisions on farms. As shown in the advantages and constraints table ( Table 5) for each practice, many technical, socio-cultural, economic, and political issues are interdependent. Therefore, linking these different levels would enable stakeholders to play a role in agroecology development. This avenue for action would make it possible to configure, enlarge, and nurture spaces of trust in which stakeholders are empowered to adjust and adapt and to think and act collectively.

### Limitations

This study had several limitations. First, we did not gather a similar number of responses for all workshops. A larger sample of responses would have been required to study more precisely certain variables (age, gender, farming systems, income) with greater statistical depth. Second, we acknowledge the potential for biased responses from workshop participants, since workshops emphasized EcoStack results on agroecological practices, attracting participants who were already interested in agroecology. This way of dissemination may have led to more responses from existing small networks of actors and specific epistemological communities, perhaps to the exclusion of other actors with fewer institutional links but with potentially greater expertise. Another limitation is that, due to the multiple workshops in the various countries, we were dependent on the data provided by project colleagues, sometimes with total and global results without the possibility of always being able to disaggregate the data (by country or participant profiles).

## Conclusion

This study was based on the results of workshops held in different European countries in 2022 and 2023. We investigated the current use of agroecological practices among participants and their approach to combining agroecological practices, also known as stacking. Our results show that participants have a relatively good knowledge of agroecological practices. Farmers have already applied for some of them, often for reasons related to legislation and national or European subsidies. However, the practice of stacking remains an approach that is difficult to explore and less attractive to stakeholders, owing to its complexity and associated uncertainties. In our view, a number of conditions need to be met to make the leap into this complexity, which would enable us to move away from an administrative approach to cultivation (legislation/subsidy) and to think more holistically at the farm level.

A successful transition pathway for agroecology requires transdisciplinary interaction, bridging science and practice, considering farmers’ practical experiences and perspectives. Better knowledge of combinations of practices, with a much larger sample of farmers and a phase of scientific and technical testing and support, followed by a cost-benefit analysis of stacking, would provide us with more comprehensive information that would allow us to prioritize certain combinations of practices that are considered more effective, socially just, and environmentally friendly than others. 

## Ethics and consent

The conditions of the study complied with ethical standards and were reviewed by The Research Ethics Committee of the University of Aarhus. The project as such, as well as the protocols for the project tasks, were accepted the 15 February 2021 (Journal no.: 2020-0185413; Approval number: 2020-104). We conducted this survey in accordance with ethical standards, and informed consent was obtained from all interviewees. Participants were adults whom we interviewed exclusively in relation to agroecological practices and not on sensitive subjects (e.g. health, sexual orientation, politics, etc.). We invited participants in advance with a presentation of the project's objectives. They were offered a consent form, which was signed by all the participants. This consent form was used to obtain their agreement to the recording of the workshops. In particular, it was indicated that “By signing the participation list, you consent to participate in the workshop and thus understand that your participation is voluntary and that all information you provide will be treated confidentially and only used by the ECOSTACK researchers for research purposes. (…) All the data gathered during the workshop will be only used for the research purposes of ECOSTACK and will be stored and used in line with GDPR. Any results of ECOSTACK will be presented only in aggregated form, so it will not be possible to identify particular workshop participants or their organisations“.

## Data Availability

For ethical reasons, the restricted data is not freely open because the raw data has not always been anonymized. The risk of reidentification is not negligible. However, any raw data used in the manuscript are available from the corresponding author ( japerrin@isara.fr) upon reasonable request. The dataset that can be transmitted is made up of various reports from workshops held in different countries during the course of the project. These reports include both quantitative and qualitative data (images/photos of the exercises carried out with participants, written descriptions of the data). A basic condition to access to this dataset will be to certify that the requester complies with the European General Data Protection Regulation (GDPR) and the data will not be used to identify individuals.
