# Supplementary material for: Farmers’ and stakeholders’ views on the adoption of agroecological practices. Results from participatory workshops in European countries
Source: Open Res Eur. 2025 Dec 11;5:180. Originally published 2025 Jul 8. [Version 2] doi: 10.12688/openreseurope.20536.2 (PMC12910197; doi:10.12688/openreseurope.20536.2)
Supplement: Peer review history

# Reviewer report: Reviewer response for version 2

**DOI:** 10.21956/openreseurope.23990.r69172

**Published:** 2026-03-31

**Authors:** Rachel Wynberg

**Affiliations:** 1 University of Cape Town, Cape Town, South Africa

This is an interesting paper, thank you for the opportunity to review it. I note that there have been several substantial reviews prior to mine so I'm not entirely clear on the value I can add but here follow a few comments:

 1. The study's objectives, abstract and content could be better aligned for improved coherence. For example, the abstract includes no mention of the study's objectives, and the focus on biodiversity in the abstract is not carried through to the objectives. The introduction (paras 3 and 4) notes a mismatch between farmer expectations and research needs, but it's not clear if this an integral part of the objectives, nor how it carried through in the discussion. These aspects need more explicit attention.

 2. I appreciate that the country selection is linked to the researchers, but it would help to justify this more strongly in terms of criteria used. European countries are hugely diverse in terms of biomes, practices, farmers, national policies, demographics, social context etc and i wanted to hear more about how the context might affect practices and perceptions. Homogenising the data unfortunately obscures this interesting contextual information.

 3. There is a conflation of agroecology and 'organic' (eg in farmer selection) which seem to be assumed to be the same thing, when we know they are quite different. It would be helpful to explain this differentiation upfront. 

 4. Given the prominence of subsidies as the rationale for uptake, it would be useful to provide the reader with more information about how they specifically work to incentivise farmers. What exactly is being subsidised and does this vary from country to country?

 5. I was confused by what was meant by the difference in understanding/perception of agroecology between researchers and farmers - and I see an earlier reviewer was similarly confused. Are you saying that farmers do not have a holistic view of their farms? This seems like a very generalized statement and I would be surprised if it were true across the board. It would be interesting to know about these differences.

 6. Statement is made that the 'literature' considers transdisciplinarity desirable for 'ethical' reasons. Could you please elaborate why?

 7. The last sentence refers to socially just practices but this is the first mention. Suggest reference to this theme is either removed or more fully integrated in the article.

 8. Lastly, I would suggest an English language edit to streamline some of the language and address grammar in parts.

If applicable, is the statistical analysis and its interpretation appropriate?

Not applicable

Is the study design appropriate and is the work technically sound?

Yes

Is the work clearly and accurately presented and does it engage with the current literature?

Partly

Are the conclusions drawn adequately supported by the results?

Yes

Are sufficient details of methods and analysis provided to allow replication by others?

Partly

Are all the source data and materials underlying the results available?

Yes

Reviewer Expertise:

agroecology policy and practice; food sovereignty; agricultural R&D; social and environmental dimensions of the bioeconomy

I confirm that I have read this submission and believe that I have an appropriate level of expertise to confirm that it is of an acceptable scientific standard, however I have significant reservations, as outlined above.

# Reviewer report: Reviewer response for version 2

**DOI:** 10.21956/openreseurope.23990.r69168

**Published:** 2026-02-17

**Authors:** Chukwuma Ume

**Affiliations:** 1 Agricultural Policy and Market Research, University of Giessen Institute for Agricultural Policy and Market Research, Giessen, Hesse, Germany

This manuscript reports on 11 participatory workshops across 9 European countries (2022–2023), part of the Horizon 2020 EcoStack project, that examined farmers’ and stakeholders’ views on adopting and stacking agroecological practices to enhance biodiversity. Using mixed methods, the study assesses current and future use, benefits, barriers, motivations, and existing practice combinations. Participants are familiar with agroecological practices such as crop rotations, cover crops, and semi-natural elements, and are interested in intercropping, organic mulches, and direct seeding. Adoption is driven by subsidies, effectiveness, ease, and cost, while barriers include knowledge gaps, funding, market constraints, and information gaps. Stacking remains complex for many, seen as isolated practices rather than integrated systems. The authors emphasize the need for transdisciplinary approaches, understanding stacking synergies, and systemic thinking at the farm level.

**Clarity of Presentation and Engagement with Current Literature – Partly**

 The manuscript is well-structured, logically organized, and generally well-written. Objectives are clear, methods are coherently explained, and results are systematically presented. Nonetheless, engagement with current literature could be enhanced in several ways: (1) the theoretical integration is somewhat limited. Concepts like transition theory, socio-technical lock-in, behavioral adoption models, or practice theory are mentioned indirectly but not extensively developed. (2) The paper's novelty in relation to existing participatory agroecology studies is not explicitly emphasized. (3) The discussion could more clearly distinguish this study from previous research on adoption and agri-environmental topics.

**Study Design and Technical Soundness – Partly**

 The workshop design suits exploratory research on perceptions and practices, using a common protocol and mixed methods. Yet several weaknesses limit robustness: 1. Sampling bias due to recruitment through project networks may favor agroecology supporters. 2. Uneven participation with only 146 of 286 actively responding. 3. Country imbalance as the exercise was only in five countries. 4. Quantitative analysis is solely descriptive. 5. No subgroup comparisons by country, farming system, age, or organic vs conventional. These limits are acknowledged, but their implications for interpretation are not sufficiently discussed.

**Replicability of Methods and Analysis – Partly**

 The manuscript would benefit from substantially greater methodological transparency to ensure scientific rigor and replicability. In particular, the workshop protocol should be described in sufficient detail (or provided as supplementary material), and the qualitative analysis procedure needs to be clearly explained, including whether coding was inductive or deductive, which software (if any) was used, how many coders were involved, and whether inter-coder reliability or validation procedures were applied. In addition, the manuscript should clarify how data were standardized and harmonized across countries, how qualitative inputs from different workshops were aggregated and synthesized, and how rankings and frequency data were processed statistically. Addressing these methodological gaps is essential to strengthen the credibility, transparency, and reproducibility of the study.

**Statistical Analysis and Interpretation – Partly**

 The statistical analysis is limited to descriptive statistics such as frequency counts and rankings, which is suitable for exploratory participatory research. However, it does not include inferential tests, cross-country comparisons, subgroup analyses, or uncertainty measures. Some percentages are interpreted substantively without adequate contextualization. Although the overall interpretation remains cautious, some conclusions go beyond what purely descriptive data can support. The manuscript should clearly state that the findings are exploratory and descriptive, avoid over-generalizations beyond the workshop sample, and, where possible, add basic comparative analyses—such as comparing organic versus conventional farmers or simple country-level differences—to enhance analytical depth without overstating the evidence.

 To ensure the scientific rigor of the study, several key issues must be addressed. First, the methodological description needs clearer explanation, especially regarding qualitative coding methods, how data was aggregated across countries, how workshop exercises were standardized, and the analytical processes used for the quantitative data. Second, the conclusions should be carefully moderated to acknowledge the exploratory and non-representative nature of the study, avoiding overgeneralizations beyond the data's empirical boundaries. Third, the manuscript should more explicitly address potential sample bias and limitations in representativeness, framing the findings as illustrative insights from workshop participants rather than definitive evidence about European farmers as a whole.

**Overall Recommendation**

 The study addresses a timely and relevant topic and provides valuable cross-country participatory insights. The design is appropriate for exploratory research, and the manuscript is generally clear and coherent.

 However, methodological transparency and analytical depth must be strengthened to ensure scientific robustness. With clearer methodological reporting, improved analytical justification, and moderated conclusions, the manuscript can become scientifically sound and make a meaningful contribution to agroecology and policy debates.

If applicable, is the statistical analysis and its interpretation appropriate?

Partly

Is the study design appropriate and is the work technically sound?

Partly

Is the work clearly and accurately presented and does it engage with the current literature?

Partly

Are the conclusions drawn adequately supported by the results?

Partly

Are sufficient details of methods and analysis provided to allow replication by others?

Partly

Are all the source data and materials underlying the results available?

Partly

Reviewer Expertise:

Agroecology; agricultural systems and sustainability transitions; farmer adoption behavior

I confirm that I have read this submission and believe that I have an appropriate level of expertise to confirm that it is of an acceptable scientific standard, however I have significant reservations, as outlined above.

# Reviewer report: Reviewer response for version 2

**DOI:** 10.21956/openreseurope.23990.r68841

**Published:** 2026-02-16

**Authors:** Harun Cicek

**Affiliations:** 1 Research Institute for Organic Agriculture (FiBL), Frick, Switzerland

This manuscript presents a valuable and timely contribution to understanding the motivations behind farmers’ adoption of agroecological practices in the EU, with a particular focus on whether—and how—these practices are combined (perhaps “bundling” is better wording than stacking). The writing is clear, the structure logical, and the research questions well-articulated.

 That said, a few key issues merit attention to strengthen the paper’s conceptual rigor and policy relevance.

 First, the authors rightly acknowledge limitations regarding sample size and representativeness. While this does not undermine the qualitative insights, it does call for caution in generalizing findings. More importantly, the manuscript would benefit from a sharper definition of what constitutes an “agroecological practice” in the EU context. Not all sustainability-oriented practices automatically qualify as agroecological—especially when considered in isolation For instance, the no-till is a good practice, but it entails, sometimes, excessive use of herbicides. The introduction should clarify how terms like “agroecology” or “agroecological practices” are operationalized in EU policy frameworks and whether ambiguities exist—both in official guidance and in farmers’ own interpretations.

 This leads to a deeper conceptual question: Does the mere bundling of multiple “sustainable” practices automatically confer an agroecological identity? Consider a no-till farmer who relies heavily on herbicides, or an apple grower using intensive pesticide regimes but also planting diverse cover crops. Such cases highlight potential contradictions that challenge simplistic categorizations. The discussion could be enriched by reflecting on these tensions—perhaps drawing on the idea of “partial” or “transitional” agroecology, where trade-offs and coexistence of conventional and agroecological elements are acknowledged.

 To further contextualize barriers to implementation, I recommend engaging with recent literature on farmer perceptions and sociocultural dimensions of agroecological transitions, particularly in Mediterranean systems. Three relevant studies include:  (Refer 1 to 3)

 These works offer nuanced insights into how tradition, identity, innovation, and policy shape on-farm decisions—dimensions that could strengthen the manuscript’s analytical depth.

 Overall, this is a strong foundation for an important discussion. With modest refinements—particularly around definitions, conceptual boundaries, and policy context—the paper could make an even more compelling contribution to the agroecology literature.

 Finally, a minor technical note: Figure 1 appears to be low-resolution. The authors might consider redrawing it as a schematic diagram to improve clarity and visual quality.

 In results section, this sentence has to “practices”: The three practices most commonly used practices (more than 10%) are currently diversified crop rotations.

If applicable, is the statistical analysis and its interpretation appropriate?

Partly

Is the study design appropriate and is the work technically sound?

Partly

Is the work clearly and accurately presented and does it engage with the current literature?

Yes

Are the conclusions drawn adequately supported by the results?

Partly

Are sufficient details of methods and analysis provided to allow replication by others?

Yes

Are all the source data and materials underlying the results available?

Yes

Reviewer Expertise:

Agroecology, agroforestry, conservation agriculture

I confirm that I have read this submission and believe that I have an appropriate level of expertise to confirm that it is of an acceptable scientific standard, however I have significant reservations, as outlined above.

## References

[1] Perceptions and sociocultural factors underlying adoption of conservation agriculture in the Mediterranean https://link.springer.com/article/10.1007/s10460-023-10495-7.

[2] Mediterranean farmers’ understandings of ‘good soil management’ and ‘good farmer’ identity in the context of conservation agriculture https://www.tandfonline.com/doi/full/10.1080/14735903.2024.2335083.

[3] A critical assessment of conservation agriculture among smallholders in the Mediterranean region: adoption pathways inspired by agroecological principles https://link.springer.com/article/10.1007/s13593-023-00926-4/metrics.

# Reviewer report: Reviewer response for version 2

**DOI:** 10.21956/openreseurope.23990.r65921

**Published:** 2026-01-07

**Authors:** Joeva Rock

**Affiliations:** 1 Stony Brook University, Nicolls Rd,, Stony Brook, USA

Thank you to the authors for taking the time to respond to reviewer comments and make revisions. The article reads much stronger now. Congratulations!

If applicable, is the statistical analysis and its interpretation appropriate?

Not applicable

Is the study design appropriate and is the work technically sound?

Yes

Is the work clearly and accurately presented and does it engage with the current literature?

Partly

Are the conclusions drawn adequately supported by the results?

Yes

Are sufficient details of methods and analysis provided to allow replication by others?

Partly

Are all the source data and materials underlying the results available?

Yes

Reviewer Expertise:

Food security, food sovereignty, anthropology of development, agricultural development, food systems, food movements

I confirm that I have read this submission and believe that I have an appropriate level of expertise to confirm that it is of an acceptable scientific standard.

# Reviewer report: Reviewer response for version 1

**DOI:** 10.21956/openreseurope.22220.r60031

**Published:** 2025-10-22

**Authors:** Joeva Rock

**Affiliations:** 1 Stony Brook University, Nicolls Rd,, Stony Brook, USA

Thank you for the opportunity to review this interesting paper on adoption and perception of agroecological practices across nine European countries.

 This is timely research and the authors do well to contextualize the study within more current events (eg farmer protests across Europe). The objectives of the study were very clear, as was the overall organization of the paper. The use of tables was effective and helped to clearly show the paper’s findings. I especially appreciated Table 8/future avenues of research, and it illustrated the complexity of the issue and the various ways it could be explored

 There are a few areas where more detail would help strengthen the paper’s overall contribution:

- Methods: Providing a few more details re: EcoStack would help contextualize the research and ground the paper better
- Methods: How and why were countries chosen?
- Methods: The authors state that they recruited participants on “the basis of convenience and professional networks.” – what exactly did this entail?
- Table 5: the different colors used in the table, especially the light green, are a bit difficult to see. Perhaps more contrasting colors could be used, or perhaps non-black colors could be bolded for contrast?
- Discussion: “There was a striking contrast in the perception of agroecology between the participants and the research teams, with differing terms of reference between science and practice.” (pg 8) – more explanation/detail here would be helpful
- Table 6: did reasons for adopting agroecological practices differ considerably across country? I’m specifically curious about “subsidies” and whether this was identified across all sites, or whether it was concentrated in a few countries.
- In general, it would be interesting to see more discussion disaggregated by country

Finally, there are two parts of the paper still in draft form and should be attended to before indexing:

- Figure 1 (pg 6) is sideways and difficult to read
- Author comments are in body of text (see bolded) on pg 11-12: “Our results show that participants have a relatively good knowledge of agroecological practices. **Perhaps we should draw on some of the text in the abstract to better link with this section and inherent understanding of their benefits**. Farmers have already applied for some of them, often for reasons related to legislation and national or European subsidies.”

If applicable, is the statistical analysis and its interpretation appropriate?

Not applicable

Is the study design appropriate and is the work technically sound?

Yes

Is the work clearly and accurately presented and does it engage with the current literature?

Partly

Are the conclusions drawn adequately supported by the results?

Yes

Are sufficient details of methods and analysis provided to allow replication by others?

Partly

Are all the source data and materials underlying the results available?

Yes

Reviewer Expertise:

Food security, food sovereignty, anthropology of development, agricultural development, food systems, food movements

I confirm that I have read this submission and believe that I have an appropriate level of expertise to confirm that it is of an acceptable scientific standard, however I have significant reservations, as outlined above.

# Response

**Published:** 2025-12-08

**Authors:** Jacques-Aristide Perrin

**Affiliations:** ISARA-Lyon, Lyon, Auvergne-Rhône-Alpes, France

We thank the reviewer for the valuable comments.  

**Comment**: Methods: Providing a few more details re: EcoStack would help contextualize the research and ground the paper better

**Answer**: We added information about the global object of the EcoStack project: “The aim of this research project is to develop ecologically, economically and socially sustainable crop production strategies via stacking of functional biodiversity. A specific objective was also to study farmer perspectives regarding a shift in focus from implementing single farm management practices to ‘stacking’ different practices. While benefits of agroecological practices are widely known, little information is available on combining those practices.”  

**Comment**: Methods: How and why were countries chosen?

**Answer**: We added: “The countries in which the workshops took place are the countries of the research teams involved in this project”.  

**Comment**: Methods: The authors state that they recruited participants on “the basis of convenience and professional networks.” – what exactly did this entail?

**Answer**: We added “All partners used a common protocol, specifying different workshop phases with several exercises as well as questions asked to participants. All participants were contacted in advance by e-mail, are…”  

**Comment**: Table 5: the different colors used in the table, especially the light green, are a bit difficult to see. Perhaps more contrasting colors could be used, or perhaps non-black colors could be bolded for contrast?

**Answer**: We added:  “There was a difference between the colors shown in the table and the actual colors. We have corrected this: While the green color corresponds to environmental services, the black color refers to the technical management of the farm, and the orange color indicates economic dimensions”.  

**Comment**: Discussion: “There was a striking contrast in the perception of agroecology between the participants and the research teams, with differing terms of reference between science and practice.” (pg 8) – more explanation/detail here would be helpful

**Answer**:  We provide now some more explanation: “There was a striking contrast in the perception of agroecology between the participants and the research teams. Indeed, the terms used by both to which the practices refer do not come from the same frame of reference between science on the one hand and the administrative context that financially supports the practice on the other”.  

**Comment**: Table 6: did reasons for adopting agroecological practices differ considerably across country? I’m specifically curious about “subsidies” and whether this was identified across all sites, or whether it was concentrated in a few countries. In general, it would be interesting to see more discussion disaggregated by country

**Answer**:  We explain a bit more the limitations:  “Another limitation is that, due to the multiple workshops in the various countries, we were dependent on the data provided by project colleagues, sometimes with total and global results without the possibility of always being able to disaggregate the data (by country or participant profiles)”.    With data from a questionnaire, it would have been easy to disaggregate it. Unfortunately, the format of the workshops, organized by different people, makes this impossible because the data was not all transmitted in the same way. We were only able to analyze the data in a limited way, which explains why it is difficult to see differences between countries and between types of participants. Furthermore, the orders of magnitude (number of responses per country or per profile) are not always equivalent.

**Comment**: Finally, there are two parts of the paper still in draft form and should be attended to before indexing: Figure 1 (pg 6) is sideways and difficult to read

**Answer**: We asked the publisher because the formatting was done by them.

# Reviewer report: Reviewer response for version 1

**DOI:** 10.21956/openreseurope.22220.r56707

**Published:** 2025-09-04

**Authors:** Yohanes Kamakaula

**Affiliations:** 1 Universitas Papua, Manokwari, Indonesia

1. The manuscript has been well-developed with strong data support. However, several sections require additional elaboration, including agroecological concepts, research objectives, methodology, and conclusions. Furthermore, literature older than 10 years should be avoided.

 2.  The technical execution of the work is commendable. However, the research design requires several improvements, particularly in the methodology section.

 3.  The research design section warrants special attention, as several components have not been comprehensively addressed, namely: research paradigm, data sources, data types, and data analysis procedures.

 4.  While the research data is of good quality, the foundational material supporting the findings requires additional exploration, particularly in the introduction, objectives, and methodology sections.

 5.  Given the nature of this research, it clearly does not require statistical analysis. Therefore, I do not have the authority to comment on this aspect.

 6.  The conclusions are adequately supported by the research findings. However, the presentation remains overly lengthy and should be more concise with brief and substantive justifications.

If applicable, is the statistical analysis and its interpretation appropriate?

I cannot comment. A qualified statistician is required.

Is the study design appropriate and is the work technically sound?

Partly

Is the work clearly and accurately presented and does it engage with the current literature?

Partly

Are the conclusions drawn adequately supported by the results?

Yes

Are sufficient details of methods and analysis provided to allow replication by others?

Partly

Are all the source data and materials underlying the results available?

Yes

Reviewer Expertise:

NA

I confirm that I have read this submission and believe that I have an appropriate level of expertise to confirm that it is of an acceptable scientific standard, however I have significant reservations, as outlined above.

# Response

**Published:** 2025-12-08

**Authors:** Jacques-Aristide Perrin

**Affiliations:** ISARA-Lyon, Lyon, Auvergne-Rhône-Alpes, France

We thank the reviewer for the valuable comments.  

**Comment**: “Furthermore, literature older than 10 years should be avoided”.

**Answer: **We removed the oldest references, sometimes replacing them with more recent ones. However, we left 2-3 older references where they refer to a particular theory or approach. Here is the list of the remoted references: ****Milestad *et al*., 2011  Defrancesco *et al*., 2008) (Toma and Mathijs, 2007)  Darnhofer, 2012) (Buckley and al., 2012)  

**Comment**: “the presentation remains overly lengthy and should be more concise with brief and substantive justifications”.

**Answer**: We shortened a bit the abstract (but added also one sentence as requested – see next comment). But see it difficult to cut more because then important points to introduce into the paper would disappear.  

**Comment**: “several sections require additional elaboration, including agroecological concepts”

**Answer**: We added these sentences: “As a set of farming practices, agroecological practices (Wezel *et al.*, 2014) and their enhanced use can play an important role in supporting these expectations in mitigating the negative environmental impacts associated with conventional or intensive agriculture and support biodiversity such as establishing hedgerows or planting flower strips”  

**Comment**: “The research design section warrants special attention, as several components have not been comprehensively addressed, namely: research paradigm, data sources, data types, and data analysis procedures.”

**Answer**: We added more information about these elements: Through this study on practices, we consider agroecology as a dynamic and holistic approach including made up of science, implementation and management of agroecological practices and transition to sustainable and fair food systems. Our work is based on a mixed-methods approach that allows us to study the interactions between agricultural practices, public policies, and social dynamics involved in the agroecological transition. This paradigm is particularly well-suited to analyzing the perceptions and experiences of farmers and advisors, and to understand how these elements influence their decisions and trajectories.  

**Comment**: improve “Methodology: data sources, data type, data analysis procedure”

**Answer: **We added more information about this: Data sources included responses to structured exercises on the adoption of agroecological practices, written workshop materials (flipcharts, ranking tables), and facilitator notes. Two main types of data were generated. First, quantitative data consisted of scoring of current and anticipated use of agroecological practices, rankings of practice combinations, and frequency counts of perceived benefits and barriers. Second, qualitative data were derived from transcribed discussions, including participants’ statements and arguments. A mixed qualitative–quantitative analytical approach was applied. Descriptive statistics were used to identify the most frequently implemented agroecological practices and practice combinations, thereby helping to characterize general trends across workshops. In parallel, a thematic content analysis was conducted to identify motivations for adopting specific practices, perceived barriers, and knowledge needs.
